# Accessory breast plasma cell mastitis managed with traditional Chinese medicine: A case report

**DOI:** 10.1097/MD.0000000000050547

**Published:** 2026-09-04

**Authors:** Yiyun Zhu, Jun Zhang

**Affiliations:** a Graduate School, Nanjing University of Chinese Medicine, Nanjing, Jiangsu, China; b Wuxi Hospital Affiliated to Nanjing University of Chinese Medicine, Wuxi, Jiangsu, China.

**Keywords:** accessory breast, axillary masses, Chinese medicine, external treatment, plasma cell mastitis

## Abstract

**Rationale:**

Plasma cell mastitis occurring in accessory breast tissue is a rare and diagnostically challenging disease. This case report aims to raise clinicians’ awareness of this condition when they encounter axillary masses, and avoid unnecessary interventions on the normal breast tissue. It also demonstrates the feasibility of traditional Chinese medicine (TCM) as a conservative treatment for patients unwilling to undergo hormone therapy or surgery.

**Patient concerns:**

A 30-year-old nulliparous woman presented with a 1-month history of a 4 × 5 cm mass in the left axilla. Empirical antibiotic therapy failed to relieve symptoms, and serological workup excluded autoimmune or infectious etiologies.

**Diagnoses:**

Ultrasound revealed hypoechoic lesions in the left accessory breast. Diagnosis was confirmed by core needle biopsy and CD138 (syndecan-1) immunohistochemistry, which showed plasma cell-predominant infiltration and ruled out granulomatous mastitis.

**Interventions:**

Due to the patient’s refusal of long-term corticosteroids, she underwent oral TCM decoction along with topical Qingbao powder. Incision and drainage were performed on pus-formed areas, followed by Jiuyi Dan powder application to promote pus discharge.

**Outcomes:**

After 2 months of treatment, the mass significantly regressed with complete symptom relief. The patient remained recurrence-free at 6-month follow-up, with only residual pigmentation, and was satisfied with the scar preservation effect.

**Lessons:**

Enhancing the recognition of early axillary masses, combined with imaging examinations and pathological biopsy to determine their nature, serves as the foundation for individualized treatment. Accessory breast plasma cell mastitis should be included in the differential diagnosis of axillary masses. TCM offers a safe, effective nonsurgical option for eligible patients, while elective surgical excision of residual accessory breast tissue is recommended to prevent recurrence.

## 1. Introduction

Plasma cell mastitis (PCM) is a nonbacterial chronic inflammatory disease characterized by periductal plasma cell infiltration and ductal ectasia. This distinguishes it from idiopathic granulomatous mastitis, which is defined by lobulocentric noncaseating granulomas. PCM is more common in young and middle-aged women.^[1]^ Its clinical manifestations include breast lumps, nipple inversion, nipple discharge, and recurrent periareolar abscesses. Due to the lack of specificity, it is often confused with breast cancer or other inflammatory diseases. Therefore, pathological examination is the gold standard for diagnosis. The disease has a prolonged course, is prone to repeated rupture and even the formation of chronic sinus tracts, seriously affecting the patients’ life quality.

Accessory breasts originate from incomplete regression of the embryonic mammary gland primordium, with an incidence rate of about 1 to 6% in the population, mostly located in the axillary region.^[2]^ A recent case series of non-lactational mastitis in accessory breast reported only 6 pathologically confirmed PCM cases among 31 patients.^[3]^ This suggests that accessory breast PCM is relatively rare in clinical practice, and there is no consensus on its diagnosis and treatment at present. This article reports a case of accessory breast PCM, aiming to explore its clinical features, diagnostic procedure, and treatment strategies in combination with this case, and enhance the awareness of clinical workers to differentiate this disease when encountering axillary lumps, so as to avoid unnecessary diagnostic and therapeutic interventions.

## 2. Report of the case

The patient is a 30-year-old female who presented with a left axillary mass accompanied by swelling and pain for 1 month. She had no family history of breast cancer or prior breast trauma, no history of childbirth, and had 2 induced abortions. The patient had no systemic symptoms such as fever, night sweats, or fatigue. She initially received empirical oral moxifloxacin hydrochloride (400 mg once daily) at a referring hospital. Although the precise duration was difficult to ascertain due to poor recollection and intermittent adherence, the treatment course was estimated to be at least 1 week. However, symptoms did not improve, and she was referred to our department.

**Figure 1.**
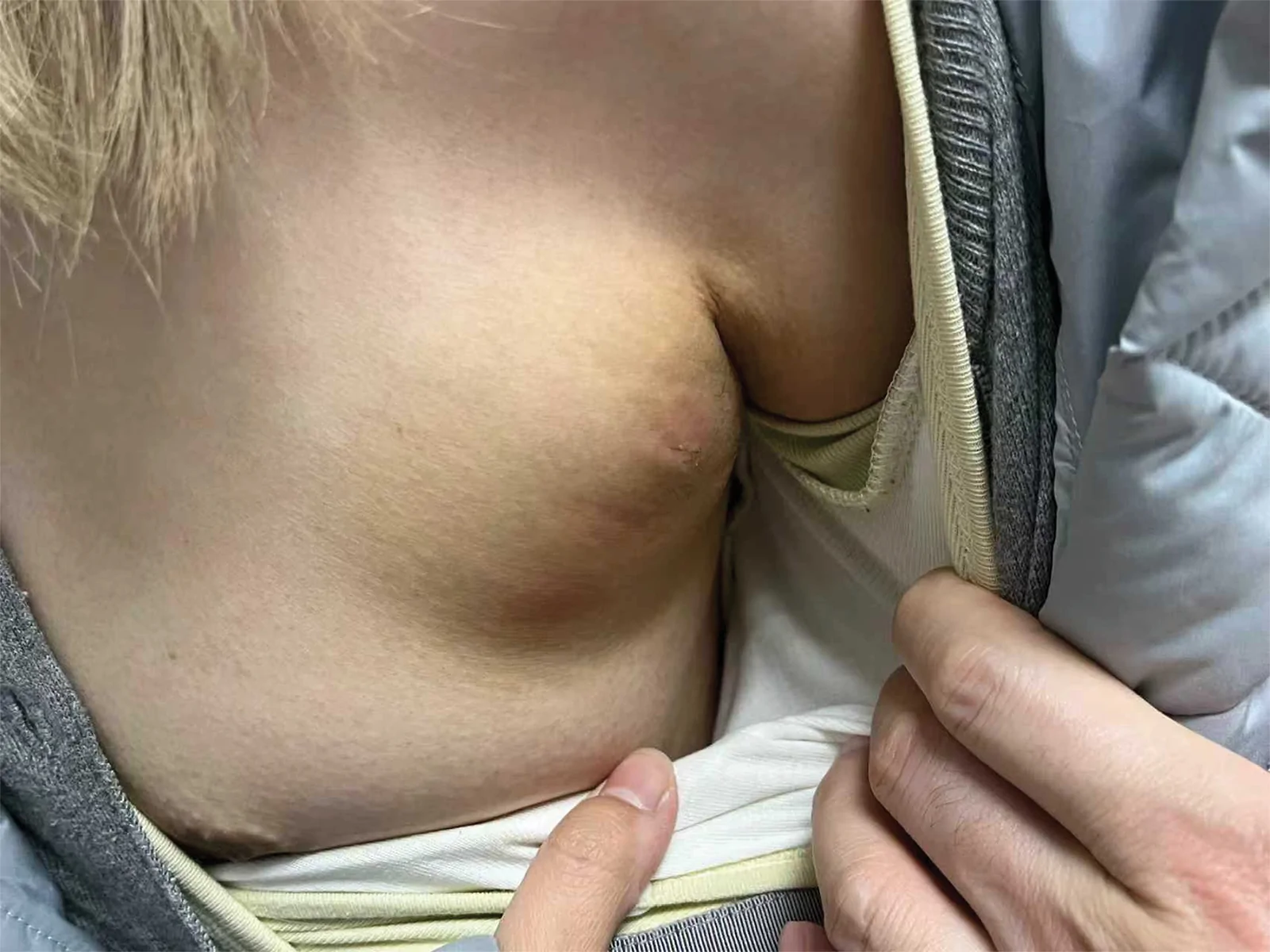
Clinical photograph of the left axillary region at initial presentation illustrating a lump with unclear boundaries, surface redness, and a size of approximately 4 x 5cm. Images of the contralateral axilla and bilateral breasts were not routinely acquired, as the clinical focus was on the symptomatic unilateral lesion, and the patient expressed discomfort with extensive photographic exposure during outpatient visits. The unaffected right axilla appeared normal upon physical examination.

**Figure 2.**
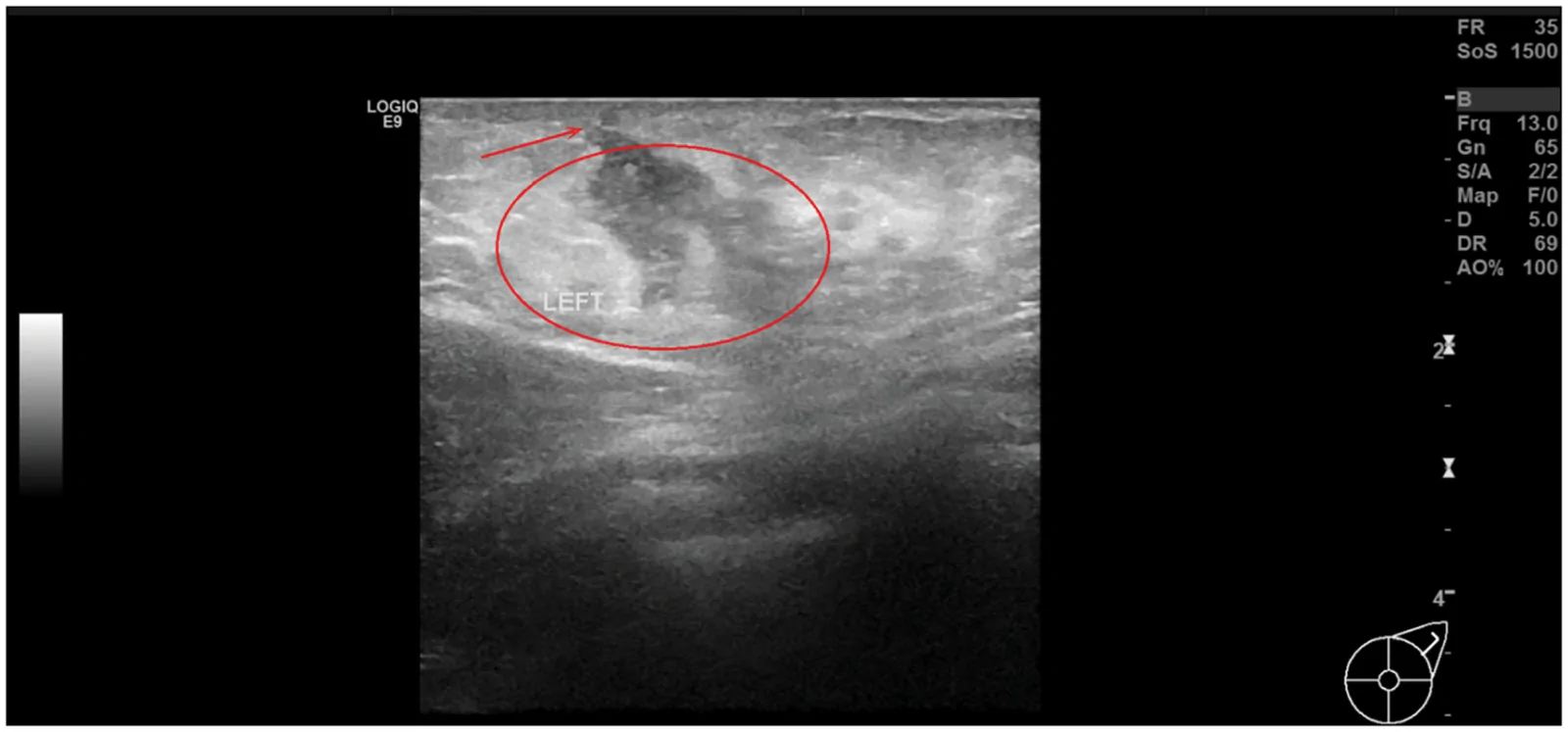
Ultrasound of left axillary accessory breast tissue at initial presentation: A breast-like structure contains patchy hypoechoic areas (red ellipse), some extending superficially toward the skin surface (red arrow).

A hard mass about 4 × 5 cm in size was palpable in the left axilla, with unclear boundaries, redness and high skin temperature on the surface, and obvious tenderness upon palpation (Fig. 1). The right axilla and bilateral breasts were unremarkable upon inspection and palpation. Scattered areas of faint erythema were visible on the skin of both lower limbs. Given her young age and high breast density, ultrasonography was prioritized over mammography. The breast ultrasound indicated no suspicious lesions in the bilateral breasts, and there was an echo of accessory breast tissue in both axillae; a classically breast-like structure in the left axilla showed multiple patchy hypoechoic areas, some extending to the body surface (Fig. 2). Laboratory tests showed an elevated white blood cell count (10.56 × 10^9^/L) and neutrophil count (7.41 × 10^9^/L), significantly elevated C-reactive protein (60.9 mg/L); the serum prolactin level was within the normal range (4.66 ng/mL).

The patient initially presented to our breast clinic in February 2024. One month later, she attended the Department of Rheumatology and Immunology for back pain. According to the referral note, the patient reported having undergone serological screening at an outside hospital prior to this visit, including rheumatoid factor, anti-cyclic citrullinated peptide antibody, antinuclear antibody, anti-double-stranded DNA, anti-Sjögren’s syndrome antigen A, anti-Sjögren’s syndrome antigen B, anti-Smith, and anti-ribonucleoprotein and T-cell Spot Tuberculosis test, all of which were negative. To further exclude seronegative spondyloarthropathy, Human Leukocyte Antigen B27 was ordered by our Rheumatology Department and was also negative. The patient had no history of psoriasis, xerophthalmia, or oral/genital ulcers. Her back pain was ultimately attributed to a prior scoliosis correction surgery.

**Figure 3.**
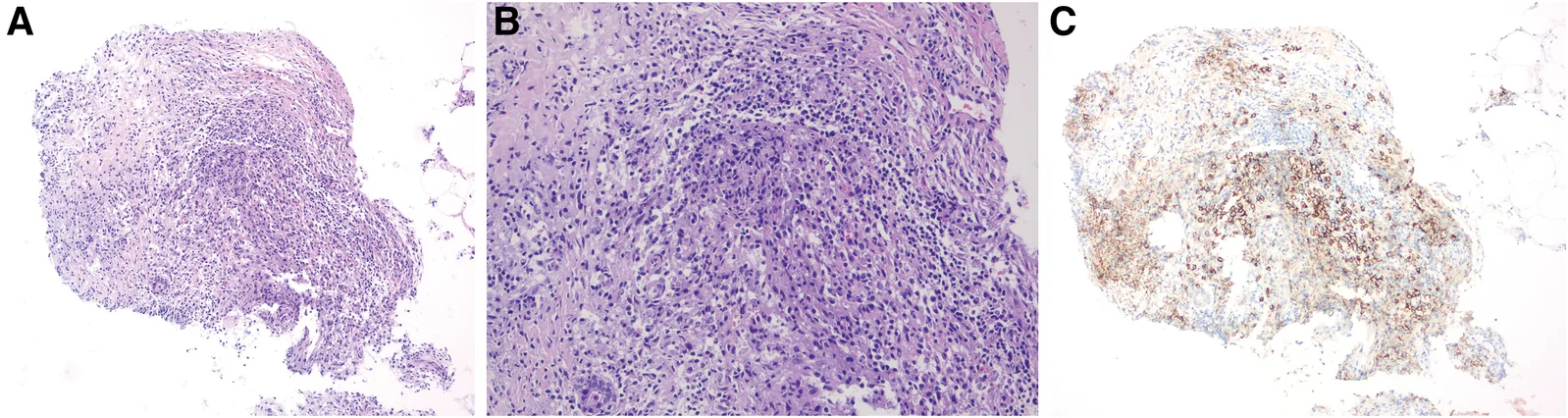
Histopathological findings of plasma cell mastitis. (A) H&E stain (×100) showing increased stromal small vessels and a dense lymphoplasmacytic infiltrate without ductal ectasia or granuloma-like structures; (B) H&E stain (×200) of the same region demonstrating the cytomorphological details, including plasma cells with eccentric nuclei; (C) CD138 immunohistochemical stain (×100) of the corresponding field showing diffuse membranous and cytoplasmic positivity, confirming the plasma cell lineage. Scale bars: A & C = 200 μm; B = 100 μm. CD138 = syndecan-1, H&E = hematoxylin and eosin.

Subsequently, a core needle biopsy was performed on the left axillary mass. Histopathological review of hematoxylin and eosin (H&E)-stained sections and additional immunohistochemical staining for CD138 (syndecan-1) were performed under the supervision of the Director of the Department of Pathology. H&E examination revealed increased small vessel proliferation in the stroma, with a dense lymphoplasmacytic infiltrate and focal neutrophilic aggregates in some areas. No ductal dilation was observed. Importantly, no epithelioid granulomas or granuloma-like structures were identified. CD138 immunohistochemical staining (×100, corresponding to the same fields as H&E) showed diffuse membranous and cytoplasmic positivity, confirming a plasma cell-predominant infiltrate (Fig. 3). Based on the combination of CD138-confirmed plasma cell predominance and the unequivocal absence of granulomatous inflammation, the diagnosis of PCM was established.

**Figure 4.**
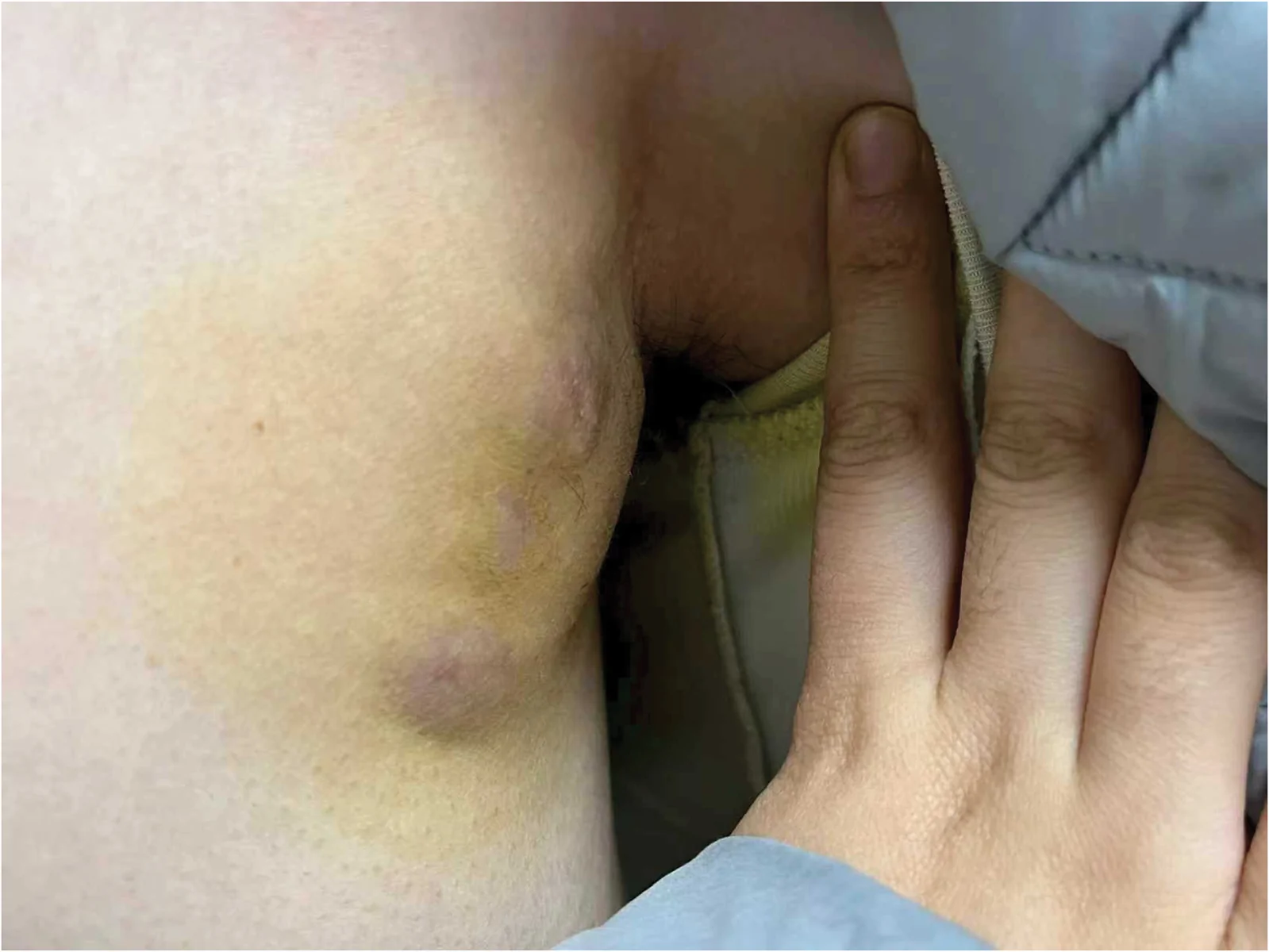
The patient’s mass in the left axilla decreased in size after incision and drainage.

**Figure 5.**
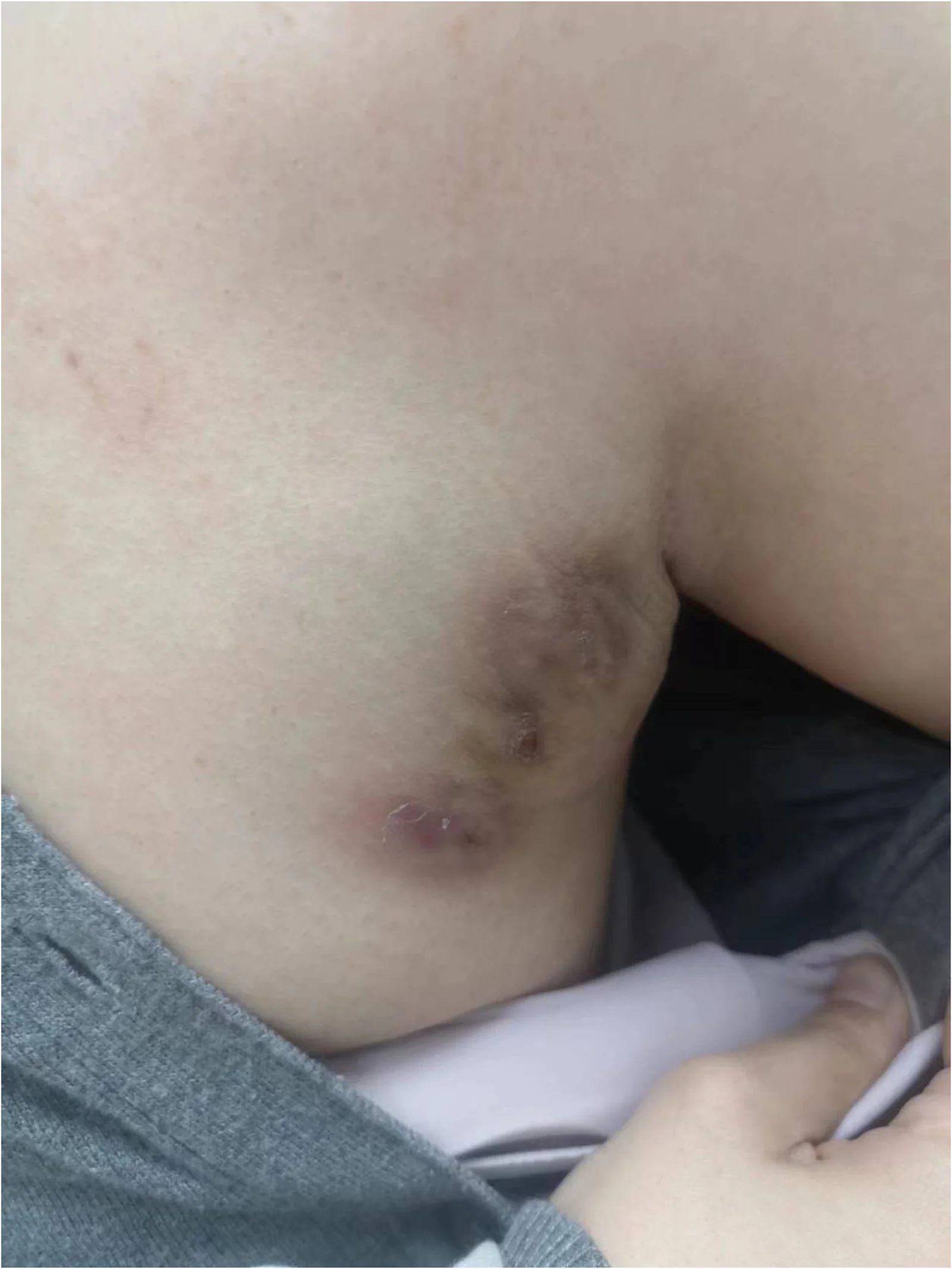
The mass in the left axilla decreased in size, and pigmentation remained.

The patient was initially treated by taking a traditional Chinese medicine (TCM) compound twice daily (200 ml each daily). Concurrently, an appropriate amount of Qinbao powder was applied daily to the affected area over the intact skin of the mass, left in place for 2 hours, and then wiped off. After 7 days of treatment, the patient returned for a follow-up visit. Physical examination revealed 3 areas of fluctuation in the left axillary mass (Fig. 4), so we performed an incision. As a result, grayish-white pus flowed out from the incision. Aerobic, anaerobic, and fungal cultures of the pus were negative. The patient continued to take the TCM decoction and inserted a piece of gauze impregnated with Jiuyi Dan powder into the incision to facilitate the drainage of the pus. After 2 months, the left axillary mass significantly shrunk and became softer. The patient reported significant relief of pain and reduction in the axillary mass. Concomitantly, the faint erythema previously noted on the lower limbs gradually faded without specific dermatological intervention. After the 6-month follow-up after treatment, only pigmentation remained in the left axilla, with no obvious mass palpable (Fig. 5). Blood routine indicators returned to normal, and no recurrence was observed. The patient reported significant pain relief, was satisfied with the scar preservation effect, and showed good compliance with TCM management. Consistent with our institution’s protocol for benign inflammatory breast lesions achieving complete response, the patient was discharged from regular clinical follow-up after the 6-month visit and has not returned for subsequent visits. Considering the risk of relapse without complete removal of the accessory breast tissue, we advised surgical excision of the accessory breast in order to prevent disease recurrence. However, the patient declined further surgery. Consequently, long-term surgical outcome data beyond 6 months are unavailable.

The composition of the TCM compound included: Trichosanthis Fructus 15 g, Fructus Arctii 15 g, Taraxaci Herba 15 g, *Dendranthema indicum* 10 g, *Forsythia suspensa* 6 g, Pericarpium Citri Reticulatae 5 g, *Pinellia ternata* 10 g, Poria cocos 15 g, *Ligusticum chuanxiong* 10 g, *Bupleurum chinense* 15 g, *Gardenia jasminoides* 10 g, *Viola yedoensis* 10 g, *Semiaquilegia adoxoides* 5 g, *Trichosanthes kirilowii* 5 g, and *Glycyrrhiza uralensis* 3 g. Decoction of these raw herbs into 200 ml, taken in 2 divided doses daily. After 4 weeks of continuous administration, the dose was tapered to once every other day for an additional 2 weeks as symptoms improved, resulting in a total oral treatment duration of 6 weeks. No adverse drug reactions like gastrointestinal upset and hepatorenal dysfunction were observed during the course.

Qingbao Powder is a preparation from Wuxi Hospital of TCM. Its composition is *Rheum palmatum*, *Phellodendron chinense* Schneid, *Curcuma longa*, *Angelica dahurica*, *Baphicacanthus cusia*, *Bletilla striata*, *Trichosanthes kirilowii*, Pericarpium Citri Reticulatae, and raw *Glycyrrhiza uralensis*. The above raw herbs are made into powder. Mix it with cold boiled water or distilled water to form a paste and apply it to the affected area. The application should extend 2 to 3 cm beyond the red and swollen area, with a thickness of 0.5 to 0.8 cm. Do this twice a day, leaving it on for 2 hours each time, and continue until the redness, swelling, and pain are gone.

## 3. Discussion

PCM is a nonspecific inflammatory disorder. It typically affects orthotopic breast tissue, while its occurrence in accessory breast tissue is relatively rare. Existing studies suggest potential causes of PCM, including ductal obstruction, smoking, and local trauma.^[4]^ Notably, accumulating evidence defines PCM as an immune-mediated disorder driven by localized dysregulation within the mammary ductal microenvironment rather than a systemic autoimmune disorder.^[5,6]^ This notion is clinically consistent with our case; despite active local inflammation in February 2024, sequential serological work-ups were negative, and the patient lacked autoimmune manifestations. The back pain was explained by prior scoliosis surgery. These findings argue against a systemic autoimmune or spondyloarthropathic origin and emphasize locally confined immune dysregulation within the accessory breast tissue.

To explain this localized pathogenesis, we propose a plausible anatomical hypothesis. Accessory breast tissue arises from incomplete regression of the embryonic mammary ridge and is characterized by structural heterogeneity, often lacking a patent ductal orifice communicating with the skin surface.^[7]^ In this case, histological evaluation revealed stromal small vessel proliferation and focal neutrophilic aggregates, reflecting an active and sustained local inflammatory response. Moreover, the absence of significant ductal dilation on histology does not rule out functional obstruction. We hypothesize that this inherent anatomical constraint likely impairs physiological drainage, leading to secretory stasis even without an increase in hyperprolactinemia. This stasis could act as an endogenous antigenic stimulus, which causes the plasma cell-predominant chronic inflammation. Importantly, the absence of epithelioid granulomas and granuloma-like structures in our sections excluded granulomatous mastitis, supporting the diagnosis of accessory breast PCM. While this hypothesis requires validation through larger clinicopathological studies or targeted imaging, it provides a coherent anatomical basis for the localized nature of the disease observed in this patient.

Ectopic breast cancer most commonly occurs in the axilla, with other sites including the vulva, chest wall, and inguinal region. Typical clinical manifestations include a localized mass, skin erythema, ulceration, and pain.^[8]^ In this case, the patient’s clinical presentation closely resembled that of axillary ectopic breast cancer, posing a significant risk of misdiagnosis and missed diagnosis, which could delay treatment. Therefore, clinicians should be on high alert when evaluating similar axillary masses. For suspected cases, prompt pathological examination is essential to confirm the diagnosis, thereby developing individualized treatment strategies as soon as possible.

Presently, no standardized guidelines exist for accessory breast PCM, as this entity remains rare. Accessory breast PCM shares similar pathological features with orthotopic PCM, so the therapeutic principles of orthotopic PCM could be logically extrapolated to accessory breast PCM. Nowadays, the treatment of PCM primarily includes conservative wound care, antibiotic therapy for superinfection, incision and drainage for abscess formation, and elective surgical resection for persistent destructive lesions.^[9]^

The patient in this case presented during the acute inflammatory phase. Performing resection of the glandular tissue and surrounding necrotic tissue at this stage could increase the risk of inflammatory spread. Therefore, we prioritized a conservative treatment approach. Given the prolonged treatment course of accessory breast PCM and the patient’s concern about potential adverse effects from long-term corticosteroid use, a combined regimen of oral Chinese herbal medicine and topical Chinese herbal application was ultimately adopted. Following sustained intervention, the patient’s mass reduced in size and softened in texture, with significant alleviation of clinical symptoms.

Compared to surgery or hormone therapy alone, Chinese medicine treatment emphasizes individualized patterns, formulating targeted strategies based on the patient’s systemic condition and local lesion characteristics. In the oral formula used for this case, the active components kaempferol from Fructus Arctii and *Gardenia jasminoides*, quercetin from *Bupleurum chinense*, and luteolin along with quercetin from Taraxaci Herba have been confirmed by modern pharmacological studies to possess anti-inflammatory and immunomodulatory effects. Specifically, kaempferol,^[10]^ quercetin,^[11]^ and luteolin^[12]^ can inhibit the nuclear factor-κB signaling pathway, downregulate the expression of pro-inflammatory cytokines such as tumor necrosis factor-α, interleukin-6, and interleukin-1β, and elevate vascular endothelial growth factor levels, thereby synergistically exerting anti-inflammatory, immunomodulatory, and tissue repair promoting effects.

Regarding Chinese medicine external treatment, in the nonsuppurative stage, Qingbao powder was applied topically. In its components, emodin and phellodendrine can inhibit edema and exudation in the early stage of inflammation,^[13,14]^ while *Bletilla striata* polysaccharides and trichosanthin demonstrate significant immunomodulatory functions.^[15]^ After suppuration and incision drainage, Jiuyi Dan (composed primarily of Gypsum and Hydrargyrum Oxydatum Crudum) was used to remove necrotic tissue and promote the growth of granulation. In traditional Chinese surgical practice, Jiuyi Dan is classified as a “corrosive-detergent” preparation: the mercury-containing component facilitates separation of necrotic slough from healthy tissue (“necrosis-removing” effect), while the gypsum matrix provides a dry, absorptive wound bed conducive to granulation tissue formation. It is important to note that due to the rich blood supply in the patient’s wound, the dosage and duration of Jiuyi Dan application must be strictly controlled and adjusted as needed. If the wound contains substantial necrotic tissue, debridement is recommended prior to Jiuyi Dan application. This approach can shorten the course of mercury-based medication, keep usage within safe limits, and simultaneously promote the drainage of pus and necrotic material, accelerating wound healing.

In summary, for patients with accessory breast PCM who are temporarily unsuitable for surgery, combined oral and topical Chinese medicine treatment is a potential initial strategy. However, in cases with significant immune dysregulation or autoimmune diseases, corticosteroids or immunosuppressants remain the cornerstone of disease management. Once inflammation is adequately controlled, elective surgical excision of the accessory breast tissue is recommended to mitigate the high risk of recurrence. Given the presence of residual accessory breast tissue bilaterally, the patient remains at risk for recurrence. We have advised regular self-examination and annual ultrasonography. Elective surgical excision is reserved if symptoms recur.

## 4. Limitations

Several limitations should be acknowledged. First of all, as a single case report, the findings are inherently observational. We still need larger cohort studies to validate our hypothesis of the pathological mechanism based on anatomy and the generalizability of TCM therapy. Secondly, we did not perform a comprehensive differential workup, including skin biopsy for the lower limb lesions and chest imaging, due to clinical constraints and the patient’s concerns. This limits definitive characterization of the cutaneous manifestations. Thirdly, the lack of long-term follow-up data limits our assessment of surgical outcomes and true disease recurrence. Finally, while TCM brought symptomatic relief in this case, its precise anti-inflammatory mechanisms remain incompletely understood. Despite these limitations, this report provides a clinically relevant account of a rare entity and a pragmatic management approach, which may inform similar cases.

## 5. Conclusion

This case emphasizes 2 principal reminders. Firstly, accessory breast PCM is a relatively rare and challenging disease. As a localized immune-mediated process, this entity may spare systemic serological markers. However, a comprehensive differential workup, including imaging and needle biopsy to exclude accessory breast cancer, targeted screening and mycobacterial culture of pus for tuberculosis and sarcoidosis, and autoimmune panels, remains essential in equivocal cases to avoid misdiagnosis. Secondly, when conventional antibiotics fail and corticosteroid use is declined because of side-effect concerns, employing TCM to resolve acute inflammation, followed by surgical excision advised as definitive treatment, is a patient-centered alternative. Due to resource and clinical constraints, we weren’t able to carry out full differential workup in this case, but future similar cases will benefit from systematic exclusion. We call for prospective multicenter studies to validate TCM-integrated therapy and inform standardized diagnostic approaches for accessory breast PCM.

## Acknowledgments

We are also grateful to all the researchers, including the physicians, nurses, and technicians, who participated in this study.

## Author contributions

**Funding acquisition:** Jun Zhang.

**Writing –original draft:** Yiyun Zhu.

**Writing – review & editing:** Yiyun Zhu, Jun Zhang.
